# Effects of multi-frequency ultrasonic assisted sodium hypochlorite on the cleaning effect and quality of fresh-cut scallion stems

**DOI:** 10.1016/j.ultsonch.2023.106613

**Published:** 2023-09-25

**Authors:** Yulan Qu, Lina Guo, Chen Hong, Yuming Wan, Jamila Tuly, Haile Ma

**Affiliations:** a School of Food and Biological Engineering, Jiangsu University, No. 301 Xuefu Road, Zhenjiang, 212013, Jiangsu, China; b Institute of Food Physical Processing, Jiangsu University, No. 301 Xuefu Road, Zhenjiang 212013, China

**Keywords:** Allicin, Cleaning processing, Fresh-cut scallion stems, Microorganism, Sweep frequency ultrasonic

## Abstract

This study aimed to evaluate the feasibility of multi-frequency ultrasound-assisted sodium hypochlorite (NaClO) on fresh-cut scallion stem (FCS) cleaning. Ultrasonic cleaning parameters (frequency mode, frequency amplitude, and the sample to water ratios) were optimized against cleanliness and microbial biomass as evaluation indexes. Under the optimum conditions, the free chlorine residues and quality attributes of FCS were also investigated. The results showed that the cleanliness of FCS improved significantly (*p* < 0.05) and the total number of microorganisms, especially *Escherichia coli*, decreased dramatically under the optimized cleaning condition with the simultaneous ultrasound (US) at the sweep frequency (SF) combination of 20 + 28 kHz, the ultrasonic density of 60 W/L, pulse time of 10 s, which indicated that the shelf life of FCS would be extended. Compared to FCS after the 250 ppm NaClO cleaning, the retention of ascorbic acid (AA), color, and texture structure of FCS had no significant difference after ultrasound-assisted NaClO treatment. Meanwhile, the content of allicin increased by 52.5% under ultrasound-assisted cleaning. The integration of US into the cleaning process resulted in a notably reduction of 68% in NaClO concentration, as well as the weight loss and respiration rate (RR) of the scallion stems. Therefore, ultrasound-assisted NaClO cleaning was regarded as a promising and effective approach for cleaning fresh-cut vegetables.

## Highlights

• Sweep frequency ultrasound (20 + 28 kHz) assisted NaClO had the best cleaning effect.
• The incorporation of US into the cleaning process decreased the use of NaClO.
• Chlorine residue in scallion stems reduced after SF-US assisted NaClO cleaning.
• The content of allicin increased under sweep frequency ultrasound-assisted cleaning.

## 1 Introduction

**Fig. 1:**
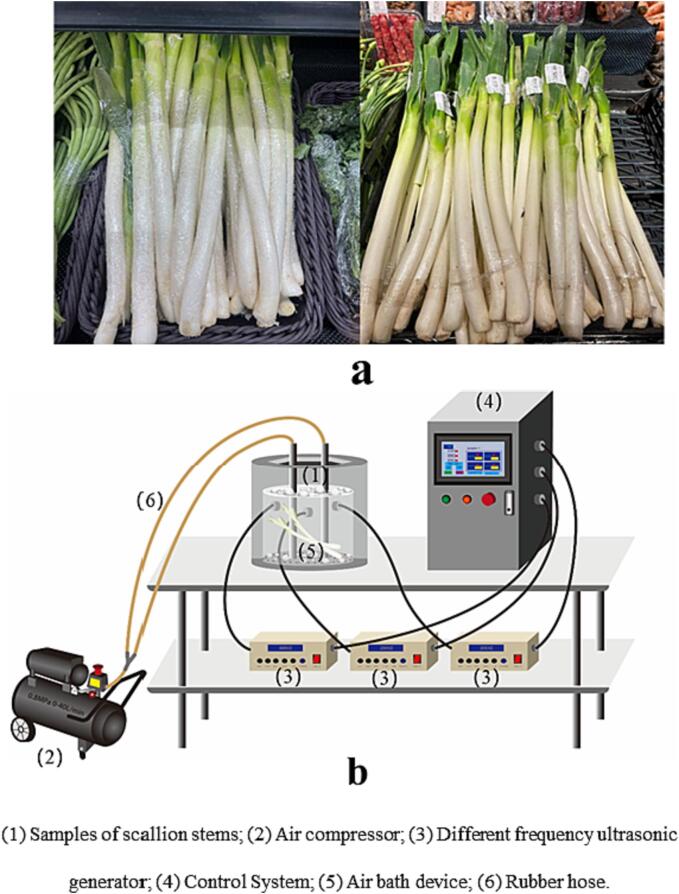
A: packaged scallion stems in the supermarket; b: Schematic diagram of ultrasonic cleaning.

Fresh-cut fruits and vegetables, also known as ready-to-eat fruits and vegetables, are safe and nutritious, which meet the nutritional needs of consumers [1]. Fresh-cut fruits and vegetables are favored by consumers with a fast-paced lifestyle due to being fresh, convenient, ready-to-eat, and ready-to-use. The scallions (*Allium schoenoprasum L.*) belong to the lily family and have long been used as a vegetable or spice in Asian countries. The scallion stem is a common fresh-cut vegetable on supermarket shelves (Fig. 1a), which contains many active ingredients, such as steroidal saponins, flavonoids, sulfur compounds, rich micronutrients, amino acids, and fatty acids [2], [3], [4]. Scallion is generally one of the main raw materials for making pickles or eaten raw. They can be contaminated during harvesting, transportation, and marketing, which causes them to carry a large number of microorganisms. Food-borne diseases have often been reported, *Salmonella* is the most common, followed by *Escherichia coli* (*E. coli*)[5], [6]. In addition, fresh-cut fruits and vegetables oozed out juices due to cut wounds, which would destroy the original ecosystem, and make it easier for microbes to grow and reproduce. Therefore, FCS has a relatively low storability. It has been well known that cleaning is an essential step to remove the soil on its surface and control the number of microorganisms on fresh-cut fruits and vegetables [7]. Meanwhile, it can clean the juice discharged from wounds on fruit and vegetables to retard food spoilage, improve edible quality, and prolong the shelf-life [8], [9]. Thus, it is crucial to adopt an effective method of cleaning and disinfection to ensure food safety.

NaClO is a traditional disinfectant widely used in tap water as well as in the disinfection of fresh fruits and vegetables. It has been reported that NaClO concentration of 400 ppm could reduce murine norovirus-1 by more than 1 log CFU/g during the washing process of Chinese cabbage and green onions, but the high concentration of NaClO also reduced the firmness of these products [10]. Similarly, NaClO has been reported to reduce total aerobic mesophilic microorganisms from 7.6 log to 5.29 log CFU/mL [11]. The industry generally requires about 10 L of disinfectant water per kilogram of fresh produce to be washed, whose concentration is commonly 250 ppm [12]. In addition, people are concerned about by-products (such as high-concentration trihalomethane) and unpleasant pungent odors produced by high levels of chlorine disinfectant [13], [14]. On twenty-seven October 2017, the International Agency for research on cancer of the World Health Organization published a list of 502 carcinogens (https://scjg.sx.gov.cn/art/2018/10/15/art_1484741_21797810.html); And the hypochlorite was on the list. Therefore, researchers are constantly searching for alternatives to chlorine disinfectants. At present, a variety of sterilization and disinfection technology has been extensively studied, including microwave sterilization, pulse sterilization, ultraviolet sterilization, ozone sterilization, and plasma water sterilization [15], [16], [17], [18], [11], [19], [20], [21], [22]. However, due to the high cost of these methods, it is difficult to recognize industrial production on a large - scale.

Ultrasound (US) is an emerging and safe cleaning approach, which is not harmful to human health or the environment. It has been broadly applied in the preservation and storage of cherry tomatoes, Chinese cabbage, strawberry, watercress, parsley, and other fruits and vegetables [23], [24], [25]. Evidence has shown that the cavitation produced by US can strip the sediment and microorganisms adhered to the surface of fruits and vegetables [26]. It has been reported that US can enhance the ability of NaClO at 200 ppm to reduce the amount of *C sakazakii* in lettuce leaves [27], which is because of the synergistic effect of ultrasound-assisted. The synergistic effect of ultrasound and NaClO have also been reported in the decontamination of fresh arugulas [28] could improve the cleaning efficiency and reduce the concentration of NaClO, such as decontamination of Chinese cabbage [10], lettuce leaves [29] and cucumbers [30], etc. The synergistic mechanism of ultrasound-assisted NaClO may be initially attributable to the potential of US-produced cavitation to generate oxidative free radicals, which can induce bacterial cell damages. In addition, the mechanical pressure generated by the ultrasound destroys the bacterial cell membrane, allowing additional NaClO-degraded hypochlorite to enter the bacterial cell interior. It leads to the burial of tyrosine and tryptophan residues and the increase of hydrophobicity [28], [31], [32], [33].

Hereby based on the aforementioned issue, this paper explored the effects of ultrasonic modes and frequency combinations on the cleaning effect and physicochemical properties of FCS in order to address the issues of poor quality and high water consumption caused by high concentrations of NaClO in the processing of cleaning fresh-cut vegetables. This study employed a feasible and efficient approach for cleaning fresh-cut vegetables to reduce surface bacteria, improve FCS storage quality, and conserve water resources.

## 2 Materials and methods

### 2.1 Raw materials and reagents

Scallions from Xinghua City, were purchased at Dongfeng Vegetable Market in Zhenjiang City, Jiangsu Province, and stored in a 4℃ dry, and light-free environment. Sodium Hypochlorite (NaClO); ethanol; sodium hydrogen phosphate; sodium dihydrogen phosphate; sodium chloride; phosphoric acid was bought from Beijing Solarbio Science & Technology Co., Ltd. (Beijing, China). All additional chemicals used for this study were acquired from Sinopharm Chemical Reagent Company (Shanghai, China).

### 2.2 Sample preparation

The sodium hypochlorite solution (effective chlorine concentration > 10%) was diluted to 100 ppm and 250 ppm with water and the concentration of chlorine was determined by the available chlorine kit. Finally, the optimum pH was adjusted to 5.5–6.0 by phosphoric acid. The scallion stems were chopped with the average length of 20 cm and diameter of 3 cm with the total weight of 1 kg. Before processing the samples, all the equipment (chopping board, knife, etc.) were soaked in the prepared NaClO solution.

### 2.3 Traditional cleaning of fresh-cut scallion stem (FCS)

The chopped scallion stems were put into treatment chamber, and then washed with different concentrations of (100 ppm and 250 ppm) sodium hypochlorite solution combined with air bubbling method. The sample was washed with air bubbling of distilled water as the control group.

### 2.4 Ultrasound-assisted sodium hypochlorite cleaning

The ultrasonic apparatus with volume of 6 L for conducting the ultrasonic experiment (Fig. 1b) was constructed by the Institute of physical processing of Jiangsu University Food Bioengineering described by Xu et al. [34] (presented in Fig. 1b). The ultrasonic tank connected to an air compressor with 0.8 MPa pressure was utilized to clean FCS. The utilization of single, dual and triple frequency of 20, 28 and 40 kHz, modes of sweep and fixed was studied. *f* is the central frequency of 20, 28, and 40 kHz. *Δf* is the amplitude of frequency, that is ± 2 kHz. When *Δf* is set to zero, it is a fixed frequency mode. 100 ppm NaClO solution was added into the treatment chamber with the total volume of 5 L. Air compressor intake was 40 L/min. To investigate the impact of various frequency and mode combinations of ultrasonic washing on the cleanliness and the microbial biomass of FCS, the experiments were carried out under multi-mode frequencies presented in Table 1 with power density of 60 W/L. Afterwards, the ultrasound-assisted NaClO cleaning was carried out by adding 100 ppm sodium hypochlorite solution into the cleaning tank at varying volume of 3, 4 and 5 L. The combination of US frequency and mode was 20 + 28 kHz sweep frequency (SF). Cleanliness and microbial reduction were used as the indicator for the screening of sample to water ratio.

**Table 1:** Screening of ultrasonic frequency and mode.

Ultrasound mode	single frequency(kHz)	dual frequency(kHz)	Tri frequency(kHz)
Fixed frequency	20,28,40	20 + 28, 28 + 40, 20 + 40	20 + 28 + 40
Sweepfrequency	(20 ± 2), (28 ± 2), (40 ± 2)	(20 ± 2)+(28 ± 2), (28 ± 2)+(40 ± 2), (20 ± 2)+(40 ± 2)	(20 ± 2)+(28 ± 2)+(40 ± 2)

Each treatment was conducted for five minutes. After treatment, samples were stored at 4 °C refrigerator in a polyethylene terephthalate container for further analysis.

### 2.5 Determination of cleanliness

For the purpose of measuring the cleanliness of the samples, the ash content of the samples was determined according to Zhao Y et al.[35]with little modification. After washing, the sample was dried at 105 °C for 2 h and then placed in an oven at 600 °C for 3 h. The samples were weighed before and after combustion. The formula for calculating ash content was as followed:

(1) $$\text{Ashes (\%) =}\;\frac{{\text{W}}_{\text{ash}}}{{\text{W}}_{\text{sample}}}\times \text{100}$$

Where W_ash_ was the weight of the final ash; W_sample_ was the initial weight of the sample.

### 2.6 Microbial analysis

The 25 g treated sample was added into 225 mL of sterilized saline solution containing the aseptic sampling bag and sealed it with a sealing machine. The sampling bag was beaten for 3 min to harvest the microbes by crushing the sample. The total number of bacteria was determined according to Zhu et al.[36], and *E.coil* was carried out according to Lin et al.[33] with minor modifications. Log microbial colony number (N) was expressed by log CFU/g. The results were expressed by microbial colony reduction logarithm (C), and the formula was as followed:

(2) $$\text{C}=log\frac{N_{0}}{N}$$

Where C is colony decrease in logarithmic value, where N is the colony number microbial colony after cleaning, and N_0_ is microbial colony on the untreated fresh sample.

### 2.7 Free chlorine analyses

Before and after cleaning, the residual content of free chlorine of samples was determined by N, N-diethyl-p-phenylenediamine (DPD) method with minor modification [37]. It was calculated using the calibration curve established with standard chlorine solution (y = 0.2407 × -0.0731 *R^2^* = 0.9995).

In brief, 10 g FCS was mixed with 10 mL 1% phosphoric acid solution shred with the mincer, and the supernatant was obtained by centrifugation for 10 min at 12000 rpm (fresh untreated samples was set as control). The absorbance was measured at 515 nm by using a T6 UV/VIS spectrophotometer (Purkinje General Instrument Co., Ltd., Shanghai, China). At the same time, distilled water was used as reagent blank instead of sample.

### 2.8 Color measurements

FCS after the cleaning was measured by a hand-held colorimeter (Hunter Associates Laboratory, Inc., America). The illuminant was D65, and the color space used was the CIE Lab system. L* stands for light/darkness; a* stands for redness/greenness; b* stands for yellowness/blueness. The color of each group was measured from 3 different points and repeated 5 times. [28]. The results are expressed by the overall color change (Δ*E*), which was calculated as follows:

(3) $$\Delta E\;=\;\sqrt{{\left(L*\;-L_{o}*\right)}^{2}+{\left(a*\;-a_{0}*\right)}^{2}+{\left(b*\;-b_{o}*\right)}^{2}}$$

where L*、a* 、b* and L_o_* 、a_o_*、b_o_* correspond to the color values of the white standard plate and treated sample before and after cleaning, respectively.

### 2.9 Texture structure analysis

The firmness of the sample was measured by a cylin drical probe with a diameter of 5 mm. Measurement conditions: trigger force 1 kg, pre-test speed and post-test speed 1.0 mm/s, test speed 0.5 mm/s, all the tests were repeated ten times, the average value was recorded [38].

### 2.10 Allicin content

The content of allicin in the FCS was quantified by the method modified from Gu et al.[39]. A total of 0.5 g of sample (dry base) was homogenized with 10 mL of 0.05 M Gibco HEPES (pH = 7.5) and incubated it at 25 ± 1 ◦C for 15 min. The extract was then centrifuged at 12,000 rpm for 5 min to determine the allicin content. After a complete reaction, the absorbance was determined at 412 nm with a photometer. The retention rate of allicin in green onions was calculated by the equations:

(4) $$C\;\left(mg/100g\right)=\frac{(A_{o}-A)\times d\times v\times 0.004}{m\;}\times 100$$

(5) $$R\%=\frac{C_{t}}{C_{o}}\times 100$$

Where C is the allicin content of the scallion stems after cleaning (mg/100 g), V is the extraction volume, m is the sample weight, d is the dilution factor, A_0_ (control) and A_t_ (samples) are the absorbance values at 412 nm, C_0_ is the allicin content of the fresh scallion stems (mg/100 g), and R is the allicin retention amount (%).

### 2.11 Ascorbic acid content analysis(AA)

The AA content of scallion stems was measured by the standard titration method using 2,6-dichloroindophenol (AOAC, 2000). The amount of AA retained was calculated by the Eqs.

(6) $${\text{C}}_{\text{vitamin c}}=\;\frac{\text{(}{\text{V}}_{\text{t}}\text{-}{\text{V}}_{\text{o}}\text{)}\times \text{T}\times \text{d}}{\text{m}\times \text{100}}$$

(7) $$R_{A}\left(\%\right)=\frac{C_{vita\min\;c}}{C_{o}}\times 100$$

Where C_vitamin_ (%) is the AA content of the sample, V_t_ is the volume consumed in the sample titration (mL), V_0_ is the volume consumed in the blank titration (mL), T is the titration, d is the dilution factor, m is the mass of the sample (g), C_0_ is AA content of fresh scallion stems (%), R_a_ is AA retention amount (%).

### 2.12 Storage period

#### 2.12.1 Weight loss analysis

After 8 days of storage at 4℃, initial weight (m_0_) for the entire storage period and the weight of at time intervals (m_1_) during the whole storage period was determined [40].The equation of weight loss rate was as follows.

(8) $$\text{Weight loss rate (\%) =}\;\frac{\left(m_{0}\text{-}m_{1}\right)}{m_{0}}\times \text{100}$$

Where m_0_ is the initial mass of the sample, g; m_1_ is the mass of the sample during storage, g.

#### 2.12.2 Respiratory rate analysis (RR)

The respiratory rate (RR) of 100 ± 10 g FCS was measured in 2 days interval in each group. After attaining a constant temperature at 25° C with the windows open and ventilated, the FCS were placed in a 5-liter container and sealed for 1 h. The respiration rate was calculated by measuring the change of gas composition within the sealed container [41]. The RR was calculated as follows:

(9) $$\text{Re}spiration\;rate\left[\mathrm{mg}/(kg\cdot h\right]\;=\;\frac{\left(C_{1}-C_{2}\right)\times V\times 1.97}{m\times t}$$

Where C_1_ is the final volume fraction (%) of the CO_2_ sealed vessel, C_2_ is the initial volume fraction (%) of the CO_2_ sealed vessel, V is the volume (mL) of the sealed vessel, 1.97 is the density (mg/mL) of CO_2_, and m is the mass (kg) of Lentinus edodes, t is the time (h) to seal the container.

### 2.13 Statistical analysis

All experiments were conducted three times, and data processed using SPSS26 software (IBM Corporation, NY, USA). Accuracy of the data was evaluated using one-way ANOVA, and Duncan test showed significant difference of each group, represented at *p* < 0.05. All values were expressed as mean ± standard deviation (SD).

## 3 Results and discussion

### 3.1 Selection of the US mode

**Fig. 2:**
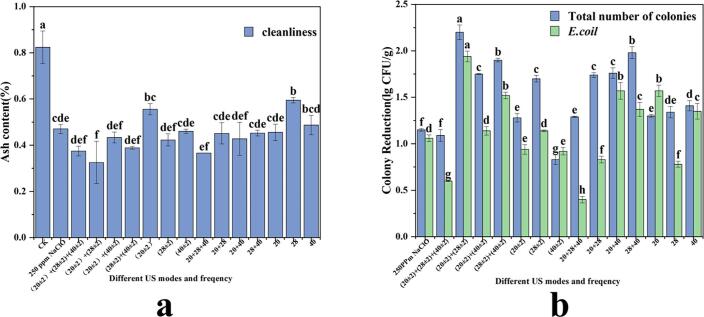
Effects of different frequency mode, frequency amplitude on the cleanliness (a) and microbial biomass (b) The results represent the means of three triplicates ± standard deviations. Different letters mean that the effects of different treatments for the same day are significantly different (*p* < 0.05).

The effect of different treatment methods on cleanness was determined by measuring the ash content of the sample. As shown in Fig. 2a. As shown in Fig. 2a, US has significantly improved the cleanliness of FCS. Under NaClO at 100 ppm, in each combination of frequency and mode (20 + 28 + 40 kHz; 20 + 28 kHz; 20 + 40 kHz and 20 kHz), the US SF has an ideal cleaning effect in cleanliness, and there was no significant difference among them (*p* > 0.05). After cleaning at a SF of (20 ± 2) + (28 ± 2) kHz US, the ash content was as low as 0.325%. Compared with the conventional NaClO cleaning of 250 ppm, the ultrasound-assisted (20 ± 2) + (28 ± 2) kHz SF with 100 ppm NaClO increased the cleanliness of FCS by 30.82%. Similar trends were reported by Zhao et al.[35], under optimal conditions (the frequency of ultrasound 40 kHz, power 180 W, sample: water = 1:100), the cleaning efficiency of edible *thelephora ganbajun* was 55.5% higher than that without US treatment. It indicated that the concentration of NaClO solution in the process of FCS cleaning could be reduced and the cleanliness of the sample could be improved by the introduction of US. Since the cavitation effect between the US and the liquid induced microbubbles to form and break while creating high pressure and temperature, it is capable of generating a huge shock wave and promote the dispersion of sediment [9].

As depicted in Fig. 2b, ultrasound-assisted cleaning under the SF of (20 ± 2) + (28 ± 2) kHz had the best effect on inhibiting microbial biomass, especially *E. coli* on the surface of scallion stems. The US treatment effect on bacterial elimination was significantly different from the conventional NaClO cleaning (*p* < 0.05). The total bacterial count reduced by 2.2 ± 0.078 log CFU/g, while the *E. coli* decreased by 1.94 ± 0.057 log/CFU, providing evidence for increase of the FCS storage duration. Previously, it was reported that the contents of mesophilic aerobes, psychotropic aerobes, molds, yeasts and three were significantly lower in the combined treatment group (28 kHz US + 10 ppm free chlorine) than the control group [42]. Similar findings were reported by Francisco et al.[28], there was a significant difference (*p* < 0.05) between ultrasound-assisted NaClO cleaning and conventional NaClO cleaning in log reduction of aerobic bacterial count on Arugula leaves. US improved the antibacterial properties of NaClO, indicating a synergistic effect on microbes’ reduction.

The above results showed that compared with other cleaning methods, the cleanliness of FCS was the best and the microbe’s reduction was the highest when the ultrasonic mode was (20 ± 2) + (28 ± 2) kHz SF. The low frequency US produced cavitation in the liquid faster and with greater force, resulting in a superior cleaning effect. In addition, the use of SF modes and alternating high and low frequencies within the same container space can reduce cavitation thresholds as well as the effects of standing wave effects [43], [44]. Thus, further experiment was carried out using a US parameter of (20 ± 2) + (28 ± 2) kHz SF.

### 3.2 The sample to water ratios screening

**Fig. 3:**
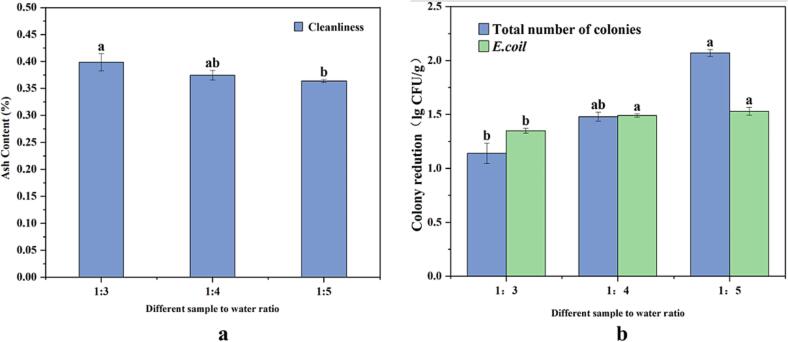
Effects of different frequency mode, frequency amplitude on the sample to water ratios on the cleanliness (a) and microbial biomass (b). The results represent the means of three triplicates ± standard deviations. Different letters mean that the effects of different treatments for the same day are significantly different (*p* < 0.05).

According to the optimum ultrasonic frequency and mode selected above, different ratio of sample to water ratios (1:3, 1:4, 1:5) were selected to for the screening experiment. The results are shown in Fig. 3a and 3b. The results revealed that ultrasound-assisted cleaning had an effect on the cleanliness and the microbial reduction (both total number of bacteria and *E.coil*), which were 0.398%, 0.374% and 0.364%；1.14 ± 0.09 log CFU/g, 1.48 ± 0.04 log CFU/g, 2.07 ± 0.03 log CFU/g and 1.35 ± 0.03 log CFU/g, 1.49 ± 0.02 log CFU/g, 1.53 ± 0.04 log CFU/g respectively (Fig. 3c). Hereby, the cleanliness of 1:4 was significantly higher than 1:3(*p* < 0.05), but non-significant with 1:5 (*p* > 0.05). Consequently, a sample-to-water ratio of 1:4 was optimal for cleaning FCS using ultrasound-assisted NaClO. The findings were consistent with those reported by Zhao et al.[35], When the ultrasonic cleaning parameters were kept constant and the material-liquid ratio reached a certain threshold, the changes in the sample-to-water ratio did not significantly impact the cleaning efficiency. This can be attributed to the thorough removal of sediment from the sample. Furthermore, the combination of ultrasonic parameters and sample to water ratio could provide the desired cleaning effect while retaining 19.99% water and 68% NaClO solution in compared to the conventional FCS cleaning technique.

### 3.3 Effect of residual free chlorine

Since free available chlorine interacts with organic molecules and generate carcinogenic byproducts of mutagens such as chloroform, chloramine, and Trihalomethane; consequently, it was vital to reduce the chance of inorganic chloride compounds employed for disinfecting vegetables [20], [37], [45]. The residual free chlorine (US + 100 ppm NaClO < 100 ppm NaClO < 250 ppm NaClO) in FCS samples washed in different methods was displayed in Table 2. The quantity of free chlorine left in the sample after ultrasound-assisted cleaning was less than the without ultrasound-assisted cleaning which was in accordance with Francisco et al.[28]. It might be due to ultrasonic-induced free radical oxidation (such as OH**^.^** and Cl**^.^**) to attack the bacterial cell body, and mechanical energy which promoted hypochlorite molecules to permeate into microbial cells. All of these above phenomena caused more free available chlorine to be used up, which led to a decline in the FCS [46].

**Table 2:** Effect of different treatment on quality of FCS.

Treatment	Residual chlorine(mg/kg)	Firmness(g)
Air bubbling	0	812.61 ± 81.567^a^
100 ppm NaClO	0.47 ± 0.027^b^	812.61 ± 81.567^a^
250 ppm NaClO	0.59 ± 0.010^c^	877.33 ± 65.214^a^
US + 100 ppm NaClO	0.27 ± 0.002^a^	887.19 ± 49.909^a^

US: (20 ± 2)+(28 ± 2) kHz sweep frequency ultrasound; NaClO: sodium hypochlorite. (Different letters indicate significant differences between treatments.) (*p* < 0.05).

### 3.4 Effect of instrument color and texture structure

Color is one of the most significant determinants of the appearance quality of fresh-cut fruits and vegetables, which has a direct influence on consumers' purchasing intention. Firmness could be described as cell wall resistance and intracellular adhesion [47], [48]. Hereby Table 2 and Table 3 were respectively revealed no significant difference in firmness and color between ultrasound-assisted NaClO treatment and traditional cleaning process (*p* > 0.05). These results demonstrated that the oxidation effects of NaClO (100 ppm and 250 ppm) and the chemical and mechanical effects of US could not alter color and firmness of FCS. Rosario et al.[30] previously reported that ultrasonic cleaning of cucumber for 5 min could improve the texture of cucumber. In this study, firmness value of FCS after ultrasound-assisted NaClO cleaning was highest (*p* > 0.05).US might have no adverse effect on the quality of FCS, and this combination of ultrasonic frequency and mode might offer the highest tissue integrity in FCS. US may have the potential to accelerate the diffusion of substances, which probably led to the homogeneity of compounds in FCS such as water and water soluble substances [49]. However, this phenomenon and its mechanism of action still needed to be better explained.

**Table 3:** Effect of different treatment on the color of scallion stems.

Treatment	L*	a*	b*	Δ*E*
Air bubbling	75.57 ± 0.906^a^	−0.14 ± 0.125^a^	10.74 ± 1.202^a^	15.92 ± 1.930^a^
100 ppm NaClO	75.40 ± 1.439^a^	−0.15 ± 0.523^a^	11.1 ± 0.832^a^	17.12 ± 2.197^a^
250 ppm NaClO	75.42 ± 1.120^a^	−0.14 ± 0.436^a^	9.25 ± 1.185^a^	17.28 ± 3.536^a^
US + 100 ppm NaClO	75.75 ± 3.089^a^	−0.15 ± 0.523^a^	10.31 ± 0.973^a^	16.61 ± 2.379^a^

US: (20 ± 2)+(28 ± 2) kHz sweep frequency ultrasound; NaClO: sodium hypochlorite. (Different letters indicate significant differences between treatments.) (*p* < 0.05).

### 3.5 Effects of different treatments on the quality of FCS

#### 3.5.1 Ascorbic acid (AA) content

**Fig. 4:**
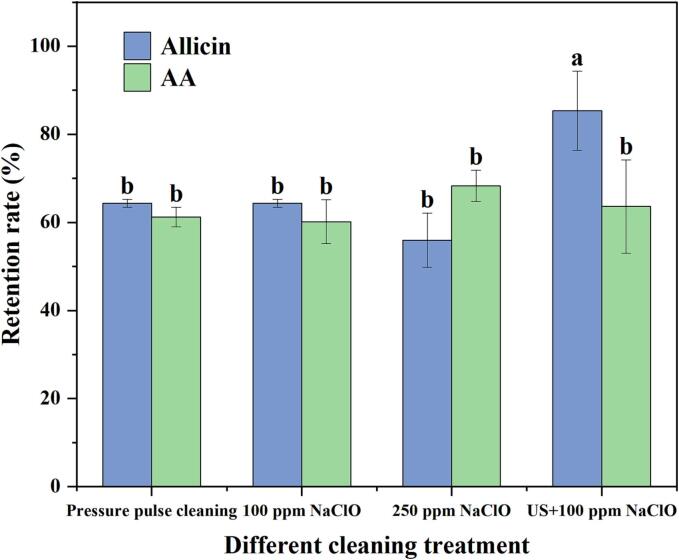
Effects of different cleaning treatment on Allicin content and AA content. (Different letters mean that the effects of different treatments for the same day are significantly different). *(p* < 0.05).

AA content is a crucial indicator to evaluate the nutritional value of fruits and vegetables, yet it was extremely unstable; and susceptible to light, heat, oxygen and water, resulting in AA loss in food processing [50]. Hence, Fig. 4 showed the retention of AA in FCS at different cleaning methods. Under all cleaning methods, the content of AA in FCS was degraded to some extent, and there was no significant difference in vitamin C retention rate among the cleaning methods (*p* > 0.05), which was inconsistent with the fingdings of Hong et al.[51]. According to their study, compared with the control group, there was no significant difference in AA content of red cabbage after ultrasonic cleaning of red cabbage until the fourth day during storage (*p* < 0.05). This was attributed to the ultrasonic frequency and mode combination that maintained the highest tissue integrity of FCS.

#### 3.5.2 Allicin content

Allicin, an important secondary metabolite in scallions, is produced by allicin and allicin enzyme during the mincing or crushing of scallions [52]sweep. Allicin has anti-bacterial and anti-inflammatory properties. Fig. 4 depicts the retention of allicin in FCS by different cleaning methods. The allicin retention rate in ultrasound-assisted NaClO cleaning was greater than that of conventional cleaning methods. The result was similar to Feng et al.[52], which might be due to the physical and chemical damage caused by ultrasonic-induced cavitation in plant cells [53]. On the one hand, that increased the chance of allicin contact with allicin enzyme, which promoted synthesis of allicin, resulting in a higher allicin retention rate. Damage of cell structure encouraged the release of allicin during the extraction process, hence the ultrasonic cleaning approach has a considerably higher allicin retention rate than conventional cleaning methods.

### 3.6 Changes of quality of FCS during storage

**Fig. 5:**
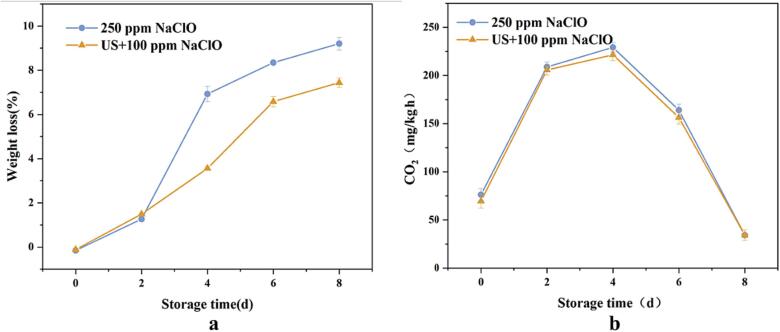
Changes in the RR (a) and weight loss (b) and of fresh cut scallion stems during storage period.

Respiratory rate (RR) was an important parameter to reflect plant life activity and the beginning of senescence. It was directly proportional to product deterioration rate and inversely proportional to shelf life. RR of each treatment group during storage period displays in Fig. 5a. It can be seen that RR of FCS was initially increased and then decreased at a certain point, and the RR reached peak on the 4th day. The results was consistent with the previous study [51]. This early stage of phenomenon might be due to the injury on the FCS caused by cutting, and later decreased of RR was because of sugar and oxygen consumption [54].

Weight has been considered as an important indicator of the post-harvest quality of fruits and vegetables, and weight loss during the storage reflected water loss and respiration rates [37], [38]. As illustrated in Fig. 5b, compared to the control group, the weight of FCS increased after ultrasound-assisted NaClO cleaning for 5 min, which was presumably because US may facilitate the mass transfer of water from a liquid medium to a sample. The lowest weight loss of FCS was 5.47% on the 8th day of storage. This might be because the cavitation of US induced hydrogen bonds between water molecules and macromolecules in FCS, resulting in the reduction of weight loss. Zhang et al.[55] have found similar results in a study of fresh cut cauliflower treated by low-frequency ultrasound cleaning during the whole storage. Moreover, the weight loss of fruits and vegetables is also associated with water loss during cellular respiratory and metabolism, so the stronger the respiration, the higher the rate of weight loss. From the above, it can be concluded that the RR of scallion stems cleaned by ultrasound-assisted NaClO was lower than that cleaned by NaClO. The change trend of weight loss also corresponded to RR.

## 4 Conclusions

In this study, we presented an approach of decontamination by sweep US of low -dual frequency combined with low-concentration chlorine, which was anticipated to offer a basis for reducing NaClO use and conserving energy. Under the optimum ultrasonic frequency and mode of (20 ± 2) + (28 ± 2) kHz dual-SF US combination and the optimum material-liquid ratio of 1:4, the ultrasound-assisted NaClO cleaning could effectively remove the surface microorganisms of FCS (The total number of bacteria decreased by 2.2 ± 0.078 log CFU/g and the total number of *E. coli* decreased by 1.94 ± 0.057 log CFU/g, which were 1.1 log CFU/g and 0.6 log CFU/g more than the total number of bacteria reduced by the enterprise production standard). Compared to the traditional FCS cleaning process, it could save 19.99% water and 68% NaClO solution. Compared to other cleaning methods, ultrasound-assisted NaClO cleaning has a higher retention rate of nutrient indexes such as ascorbic acid and allicin in scallions, along with enhanced firmness values, while exerting no adverse impact on color index. The application of US in fresh-cut fruit and vegetable processing would be profitable to post-harvest sector.

## CRediT authorship contribution statement

**Yulan Qu:** Data curation, Writing – original draft, Investigation, Validation. **Lina Guo:** Formal analysis, Writing – review & editing. **Chen Hong:** Methodology. **Yuming Wan:** Methodology. **Jamila Tuly:** Writing – review & editing. **Haile Ma:** Conceptualization, Resources, Supervision.

## Declaration of Competing Interest

The authors declare that they have no known competing financial interests or personal relationships that could have appeared to influence the work reported in this paper.
